# The Endoplasmic Reticulum–Plasma Membrane Tethering Protein Ice2 Controls Lipid Droplet Size via the Regulation of Phosphatidylcholine in *Candida albicans*

**DOI:** 10.3390/jof10010087

**Published:** 2024-01-22

**Authors:** Ying Deng, Hangqi Zhu, Yanting Wang, Yixuan Dong, Jiawen Du, Qilin Yu, Mingchun Li

**Affiliations:** Key Laboratory of Molecular Microbiology and Technology, Ministry of Education, Department of Microbiology, College of Life Sciences, Nankai University, Tianjin 300071, China; dy19881702071@163.com (Y.D.); zhuhangqi0922@163.com (H.Z.); ylgzwyt@163.com (Y.W.); dyx178606206862021@163.com (Y.D.); dujiawen0919@163.com (J.D.);

**Keywords:** lipid droplet, endoplasmic reticulum–plasma membrane tethering protein, phosphatidylcholine, Ice2, *Candida albicans*

## Abstract

Lipid droplets (LDs) are intracellular organelles that play important roles in cellular lipid metabolism; they change their sizes and numbers in response to both intracellular and extracellular signals. Changes in LD size reflect lipid synthesis and degradation and affect many cellular activities, including energy supply and membrane synthesis. Here, we focused on the function of the endoplasmic reticulum–plasma membrane tethering protein Ice2 in LD dynamics in the fungal pathogen *Candida albicans* (*C. albicans*). Nile red staining and size quantification showed that the LD size increased in the *ice2*Δ/Δ mutant, indicating the critical role of Ice2 in the regulation of LD dynamics. A lipid content analysis further demonstrated that the mutant had lower phosphatidylcholine levels. As revealed with GFP labeling and fluorescence microscopy, the methyltransferase Cho2, which is involved in phosphatidylcholine synthesis, had poorer localization in the plasma membrane in the mutant than in the wild-type strain. Interestingly, the addition of the phosphatidylcholine precursor choline led to the recovery of normal-sized LDs in the mutant. These results indicated that Ice2 regulates LD size by controlling intracellular phosphatidylcholine levels and that endoplasmic reticulum–plasma membrane tethering proteins play a role in lipid metabolism regulation in *C. albicans*. This study provides significant findings for further investigation of the lipid metabolism in fungi.

## 1. Introduction

Lipid droplets (LDs) are conserved organelles found in almost all eukaryotes. They have a special structure with a hydrophobic core of neutral lipids coated by a phospholipid monolayer with a set of proteins [1]. LDs play important roles in cellular metabolism and signal transduction, store triacylglycerols (TGs) and sterol esters (SEs) for metabolic fuel and membrane synthesis, are involved in protein transport and immunity [2], and are connected to metabolic diseases such as diabetes and atherosclerosis [3]. LDs originate in the endoplasmic reticulum (ER) and bud to the cytoplasm after reaching a certain size. Further LD growth occurs through LD fusion or by obtaining lipids from the ER and LD surfaces where lipids are produced by lipid synthase. These growth events are regulated by proteins on the LD’s surface and the connection between ER and LDs, and the phospholipid composition of the LD surface monolayer is crucial [4,5,6,7].

LD sizes vary from 100 μm to 100 nm depending on different cell types. Most cells control the size and number of LDs to within a certain range to maintain the balance of lipid consumption and storage and satisfy the needs at different stages [8]. Abnormal LD sizes affect adipogenesis, the lipolysis rate, and fatty acid oxidation [9]. The mechanism of LD size regulation is poorly understood, but previous relevant studies can provide some insights. It has been reported that phospholipids are important regulators of LD size. Phospholipids on the LD surface function as surfactants to maintain LD stability, and changes in the phospholipid composition and content may have a profound effect on LD morphology [10]. For example, large LDs are commonly accumulated in mutants with a deficiency in the phosphatidylethanolamine N-methyltransferase (PEMT) pathway, which converts phosphatidylethanolamine (PE) to phosphatidylcholine (PC). These mutants include the deletion stains of the methyltransferases Cho2 and Opi3, and the transcriptional activators Ino2 and Ino4 [11]. The LD morphology can be restored by adding the PC precursor choline [9], highlighting the role of PC in the regulation of LD size. In fact, as a significant cylindrical-shaped lipid, PC can provide a considerably lower surface tension and is considered as an important factor in LD fusion resistance [10]. Phosphatidic acid (PA) has the opposite impact on LD size. Most mutants with “supersized” LDs have elevated PA levels, including *cho2*Δ/Δ, *opi3*Δ/Δ, *ino2*Δ/Δ, *ino4*Δ/Δ, and the CDP-diacylglycerol synthase-deficient mutant *cds1*Δ/Δ (which cannot consume PA for phospholipid synthesis). Inositol addition and the overexpression of the PA phosphatases (*PAH1* and *DPP1*) to reduce the cellular PA pool were found to largely reduce the formation of large LDs in the mutants with “supersized” LDs [9,12]. From studies of PA properties, it has been suggested that PA regulates LD size by promoting LD fusion based on its effect on membrane curvature as a conical lipid [9,13,14].

Some proteins are also linked to LD size regulation. Proteins involved in TG and SE synthesis (such as Dga1 in yeast) and lipolysis (such as Tgl3 in yeast) can regulate LD growth directly [15,16]. The fat-specific protein Fsp27 can mediate LD fusion to promote LD size [17], while the integral ER membrane protein FITM1/2 enhances TG partitioning into LDs [18]. Seipin is another regulator of LD size, which is conserved in various species. It was found to be involved in the coupling of neutral lipid synthesis with LD assembly at ER-LD contact sites [19]. From humans to *Drosophila*, seipin knockout can make LDs larger and more numerous [20,21,22]. In yeast, the human seipin ortholog Fld1 forms a complex with Ldb16 at ER-LD contact sites to regulate LD size. The interaction between Fld1 and Ldb16 is crucial for maintaining normal-sized LDs; any deficiency in the interaction region can cause supersized and clustered LDs. The overexpression of Ldb16 alone can also lead to larger but fewer LDs [20].

Tethering proteins are a set of proteins that help to establish contact sites between organelle membranes; these contact sites are thought to form platforms for the exchange of lipids, metabolites, and ions and the regulation of organelle division, trafficking, and inheritance [23,24,25]. It has been reported that some tethering proteins participate in LD-related regulation, including the regulation of LD fusion and growth [17,26], LD size changes [27], and LD protein targeting [19]. Most of them are proteins that function in the attachment of LDs to other organelles. However, the contact between the endoplasmic reticulum (ER) and the plasma membrane (PM) has barely been studied for its role in LD morphology and function regulation. In our study reported here, we show that an ER–PM tethering protein, Ice2, regulates LD size in *C*. *albicans*, presenting a new way to elucidate the mechanism of lipid metabolism regulation.

## 2. Materials and Methods

### 2.1. Fungal Strains and Culture Conditions

The strains, plasmids, and primers used in this study are listed in Tables S1–S3 in the Supplementary Materials.

All strains were generated from the initial *C*. *albicans* strain WT (BWP17) with the PCR homologous recombination method or the transformation of plasmids, which were digested with a single enzyme. In order to obtain the *ice2*Δ/Δ mutant, the pRS-ARG4Δ*Spe*I plasmid was used as the template, and the knockout primers ICE2-5DR and ICE2-3DR were used to obtain the *ice2*::*ARG4* fragment, which was transformed into the BWP17 strain. Then, another cassette *ice2*::*URA3*-*dpl200* was amplified from the pDDB57 plasmid and transformed to the heterozygote to obtain the homozygous strain *ice2*::*ARG4*/*ice2*::*URA3*-*dpl200*. During these two processes, ICE2-5DET and ICE2-3DET were used to verify the properties of the transformants. Finally, a synthetic complete (SC) medium containing 5-fluoroorotic acid was used to select strains without the *URA3* marker. The construction of the complement strain and the localization strains was based on the construction of the complement plasmid and the localization plasmids. The plasmids were then linearized with single enzyme digestion and transferred to the BWP17 or *ice2*Δ/Δ strain.

*C*. *albicans* cells were grown in YPD culture medium (yeast extract 1% (*w*/*v*), peptone 2% (*w*/*v*), glucose 2% (*w*/*v*), 80 μg/mL uridine) or SC medium (yeast nitrogen without amino acids 0.67% (*w*/*v*), glucose 2% (*w*/*v*), complete amino acid mixture 0.2% (*w*/*v*), 80 μg/mL uridine) at a constant temperature of 30 °C and 160 rpm unless otherwise specified. For hyphal induction, cells were cultured in RPMI 1640 (RPMI-1640 1.04% (*w*/*v*), MOPS 0.418% (*w*/*v*), Na_2_CO_3_ 0.2% (*w*/*v*), 80 μg/mL uridine, pH 7.4, sterilization by filtration), M199 (M199 0.95% (*w*/*v*), HEPES 3.57% (*w*/*v*), 80 μg/mL uridine, pH 7.0, sterilization by filtration), or spider media (mannitol 1% (*w*/*v*), nutrient broth 1% (*w*/*v*), K_2_HPO_4_ 0.2% (*w*/*v*), 80 μg/mL uridine, pH 7.2) at 37 °C.

### 2.2. Nile Red Staining and Fluorescence Microscopy

LD staining with Nile red was performed as reported before [28]. Cells were cultured in YPD medium overnight and then transferred to SC medium (with 1 mM choline or without choline), in which the OD_600_ was adjusted to 0.1. The cells were cultured for 6 h or 12 h at 30 °C, washed twice with PBS, and then resuspended in 1 mL PBS. Nile red was prepared as a 1 mg/mL stock solution with acetone as the solvent, and 10 μL of the stock solution was added to 1 mL of cell suspension. The cell suspension was then incubated at 30 °C for 30 min. After washing with PBS 3 times, the cells were imaged using a fluorescence microscope (Olympus, Tokyo, Japan) equipped with a UPlanFL × 100 oil objective lens (Olympus, 1.3 NA) and a digital single-lens reflex camera (Canon, Tokyo, Japan). In a sequential scanning using a white light laser, the Nile red signal was acquired using a diode laser at 405 nm excitation, and the emission was collected between 420 and 480 nm. Images were analyzed with Image J 1.53t. To calculate LD size, the diameter of more than 300 LDs was measured for every sample; all LDs were chosen randomly. For the observation of GFP- or RFP-labeled proteins, various localization strains were cultured in SC medium. The cells were then collected at different time points according to demands and observed with a fluorescence microscope after 2 washes with PBS. The GFP was excited at 488 nm, and the emission signal was collected between 494 and 562 nm; mCherry was excited at 587 nm. and the emission was collected between 594 and 719 nm.

### 2.3. Triacylglycerol (TG) Level Analysis

Cells cultured in SC medium for 6 h and 12 h were collected and washed twice with sterile water before cell lysis. After the addition of RIPA lysis buffer (0.05 M Tris-HCl, 0.15 M NaCl, 0.5% sodium deoxycholate, 0.1% SDS, 3 mM EDTA) and glass beads, the mixture was violently shaken and placed in an ice-bath. This was repeated about 10 times for 2 min each. The mixture was centrifuged at 12,000 rpm for 25 min; the supernatant was used for TG measurements. TG measurements were based on the method detailed in the TG measurement kit (Nanjing Jiancheng Bioengineering Institute, Nanjing, China). In detail, 1 mL of working fluid was mixed with 10 μL of cell lysate and incubated at 37 °C for 30 min. The absorption of the mixture at 500 nm was then detected with a microplate reader (PerkinElmer LLC, Boston, MA, USA). The final TG concentration is normalized by the protein concentration, which was determined using a BCA protein assay kit (Solarbio, Beijing, China).

### 2.4. Phosphatidylcholine Level Analysis Using Thin-Layer Chromatography

Total lipids were extracted following a previously reported method with little modification [29]. In brief, the fungal cells were cultured for 12 h in 250 mL of SC medium, collected using centrifugation, washed with sterile water, and then suspended in 3 volumes of chloroform/methanol 2:1 *v*/*v* with 1 volume of glass beads. The suspensions were vortexed at high speed for 5 min followed by shaking for 1 h. Then, an equal volume of 0.9% NaCl was added. The mixture was briefly vortexed and was further centrifuged at 12,000 rpm for 10 min. The lower chloroform phase was recovered and then dried with nitrogen flow at room temperature. The final extract was dissolved in 400 μL chloroform/methanol 2:1 vol/vol for TLC analysis. For phospholipids separation, a chromatography solution containing chloroform/ethylacetate/acetone/isopropanol/ethanol/methanol/water/acetic acid at 30:6:6:6:16:28:6:2 (*v*/*v*) was prepared, and 5 μL of the sample was separated on silica gel 60 plates. The bands were stained with iodine vapor in an airtight tank and quantified using Image J 1.53t [19].

### 2.5. Detection of the Morphogenetic Ability and Drug Sensitivity of C. albicans

For liquid hyphal induction, the initial OD_600_ of cells was adjusted to 0.1, and cells were cultured in RPMI 1640 medium at 37 °C for 4 h before microscopic observation. For solid hyphal induction, the initial OD_600_ of the cells was adjusted to 0.5, and 3 µL of the cell solution was placed in the RPMI 1640, M199, and spider solid media and incubated at 37 °C for 5 d. To test the adhesion ability of *C. albicans*, cells activated overnight were washed once with sterile water and re-suspended in the liquid medium of RPMI 1640 medium, and the initial OD_600_ was adjusted to 0.1. Then, the cell solution was added to a 24-well plate and incubated at 37 °C for 3–4 h. Next, the medium was removed from the plate and washed with PBS. Then, 1% (*w*/*v*) crystal violet was added for staining; after staining for 5 min and washing with PBS, the sample was divided into two. One part was combined with 10% (*w*/*v*) acetic acid and oscillated slowly for 10 min, and the supernatant was removed to detect the OD_595_. The other part was combined with methanol to fix cells for microscopic observation.

To test drug sensitivity, cells were cultured to the logarithmic stage and collected with centrifugation. After washing with sterile water, the OD_600_ of cells was adjusted to 0.2, then the cell solution was diluted 10-fold for 4–5 gradients. Finally, 2 μL of the diluted cell solution was placed on a plate with drug. After 2–3 d of culture at 30 °C, the growth status condition of different strains was observed.

### 2.6. LD Autophagy Assessment

LD autophagy was assessed by the degradation of LD marker protein Erg6-GFP [30], and Erg6-GFP degradation was observed with Western blotting. Before Western blotting, cells were cultured in SC medium for 5 d, then the cells were collected and physically broken to release cellular components. The total cytosolic proteins of cells were prepared using vortexing and centrifugation. Both Erg6-GFP and free GFP in the samples were detected using a GFP monoclonal antibody, and Tubulin was detected using an α-Tubulin monoclonal antibody as the endogenous reference. Image J 1.53t was used to quantify the intensity of the bands of Erg6-GFP and free GFP.

### 2.7. Statistical Analysis

All experiments were repeated at least three times; the values are presented as means ± s.d. A significant difference between the treatments was determined using a Student’s *t*-test or a one-way ANOVA (*p* < 0.05). For percentage comparisons, the chi-square test was used to analyze the differences. Statistical analyses were performed using Statistical Packages for the Social Sciences (SPSS, version 20).

## 3. Results

### 3.1. Ice2 Is an ER–PM Tethering Protein in C. albicans

**Figure 1:**
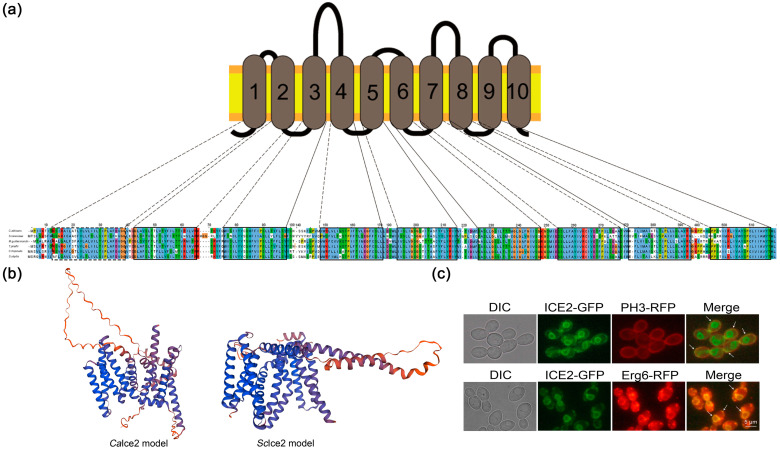
Ice2 in *C. albicans* has a conserved structure and localization. (**a**) *Ca*Ice2 has a conserved transmembrane domain. Sequences were aligned using Clustal Omega, and the transmembrane domain was predicted with TMHMM2.0. (**b**) Three-dimensional structural models of *Ca*Ice2 and *Sc*Ice2. The structural models were predicted using SWISS-MODEL. For the *Ca*Ice2 model: UniProt number of the template: A0A1D8PT87; GMQE (global model quality estimate): 0.69; Parameters: use default parameters. For the *Sc*Ice2 model: UniProt number of the template: P40499; GMQE (global model quality estimate): 0.77; Parameters: use default parameters. (**c**) Co-localization of Ice2-GFP and the PM marker PH3-RFP (upper, indicated by the arrows) in the WT + Ice2-GFP PH3-RFP strain, and co-localization of Ice2-GFP and the ER marker Erg6-RFP (lower, indicated by the arrows) in the WT + Ice2-GFP Erg6-RFP strain [33].

In *Saccharomyces cerevisiae* (*S*. *cerevisiae*), Ice2 is an ER protein that has been confirmed to participate in establishing contact sites between the ER and the PM. Ice2 deficiency with other ER–PM tethering proteins can have a severe impact on lipid biosynthesis, sterol transport, and PM stability [31]. To find the homolog of *S*. *cerevisiae* Ice2 in *C*. *albicans* and explore its function, we first performed a homology search using blast and found an unidentified homology in *C. albicans* with 33.8% identity, and we named it *Ca*Ice2. Next, we analyzed the conserved domain of *Ca*Ice2 and found that it belongs to the *ICE2* superfamily with a conserved Ice2 domain (Figure S1a). Based on the predictions of the transmembrane domain and sequence alignment, we found that Ice2 has a conserved transmembrane domain (Figure 1a and Figure S1b). Further 3D structural prediction showed that it has a similar transmembrane structure and lipid binding loop to Ice2 of *S. cerevisiae* (Figure 1b), but there is a larger ring composed of random curlings and more transmembrane helices in the *Ca*Ice2 model.

All these results suggest that *Ca*Ice2 may have similar functions to Ice2 in *S*. *cerevisiae*. Then, we observed the ER morphology of WT and *ice2*Δ/Δ and found that there were some cortical ER deficiencies in *ice2*Δ/Δ, which is consistent with the phenotype caused by *ICE2* deletion in *S*. *cerevisiae* [32]. (Figure S2). Further localization identification revealed that *Ca*Ice2 colocalizes with PM protein PH3 and ER protein Erg6 (which can also localize to LDs) at the ER–PM contact sites, suggesting that it is an ER–PM tethering protein similar to Ice2 in *S*. *cerevisiae* (Figure 1c).

### 3.2. Deletion of ICE2 Leads to the Accumulation of Large Lipid Droplets

**Figure 2:**
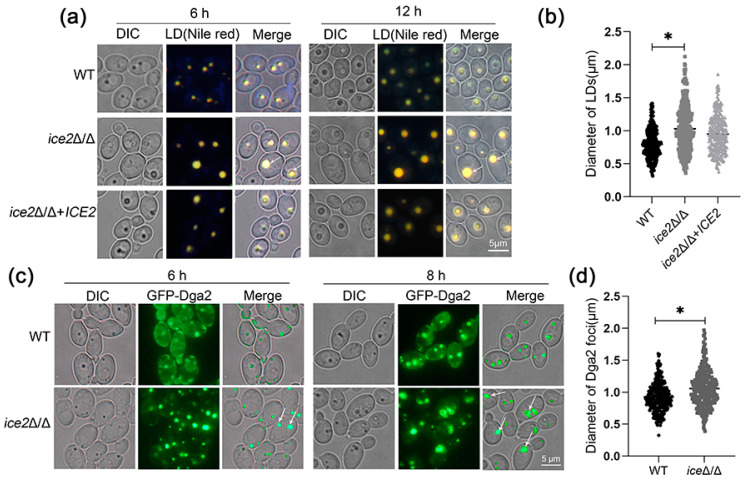
Large LDs accumulated in *ice2*Δ/Δ. (**a**) The LDs in WT, *ice2*Δ/Δ, and *ice2*Δ/Δ + *ICE2* cells grown in SC medium were stained with Nile red for fluorescence microscopy. The white arrowheads denote large LDs. (**b**) Diameter of LDs from WT, *ice2*Δ/Δ, and *ice2*Δ/Δ + *ICE2* cells grown in SC medium for 6 h. LD diameter was measured with Image J 1.53t, and for each strain, at least 300 LDs were measured. *, *p* < 0.05. (**c**) The localization of the LD marker GFP-Dga2. WT and *ice2*Δ/Δ cells were grown in SC medium for microscope fluorescence microscopy. The white arrowheads denote large Dga2 foci. (**d**) The diameter of GFP-Dga2 foci in WT, *ice2*Δ/Δ, and *ice2*Δ/Δ + *ICE2* cells grown in SC medium for 6 h. The diameter of GFP-Dga2 foci was measured via Image J 1.53t and for each strain, at least 300 foci were measured. *, *p* < 0.05.

As previous reports indicated that a deficiency of all ER-PM tethering proteins leads to abnormal lipid synthesis and sterol transport, we wondered if Ice2 could regulate lipid metabolism alone. Considering that LDs are central to lipid metabolism in cells, we first focused on the LD morphology of *ice2*Δ/Δ. Since the YPD medium with abundant nutrients could cover the possible changes in LDs [9], a SC medium was used to culture cells for LD observation. To stain LDs, the neutral lipid dye Nile red was used; further observation using fluorescence microscopy suggested that some very large LDs appeared in *ice2*Δ/Δ, which were barely observed in WT cells (Figure 2a,b). For more comprehensive and accurate observation of the LDs, we marked LDs with the GFP-labeled LD protein Dga2 (homologous to Dga1 in *S*. *Cerevisiae*, which moves from the ER to the LD surface to supply TGs for LDs and is a key factor in LD formation) [34,35]. Just as reported in *S*. *cerevisiae*, Dga2 in *C. albicans* was distributed on the ER and the LD surfaces (there appeared to be some foci), and the Dga2 foci also exhibited the accumulation of large LDs in *ice2*Δ/Δ (Figure 2c,d). To figure out whether LD sizes are related to cell sizes, we measured the cell sizes of WT, *ice2*Δ/Δ, and *ice2*Δ/Δ + *ICE2* after the cells were cultured in SC medium for 6 h and 12 h. The results showed that the deficiency of *ICE2* had no impact on cell sizes at 12 h and had a little effect at 6 h, suggesting that there was little correlation between the LD sizes and the cell sizes (Figure S3). Since the WT LDs became larger with the increase in culture time, this leads to the question of whether the large LD phenotype observed in *ice2*Δ/Δ is due to the different development stages. Therefore, we plotted the growth curve. The results showed that the tested strains entered the log phase after 6 h of culturing (Figure S4), suggesting that the three strains were in the same developmental stage during LD formation, excluding the influence of the growth stage on LD size. As the mycelial form is important for *C. albicans*, we also observed the LDs in mycelia. The results revealed that the *ICE2* deficiency led to increased LD aggregation in mycelia (Figure S5) in contrast to the yeast form, indicating other unknown mechanisms functioning in LD regulation in mycelial cells.

### 3.3. Deletion of ICE2 Impairs the Synthesis of Phosphatidylcholine

**Figure 3:**
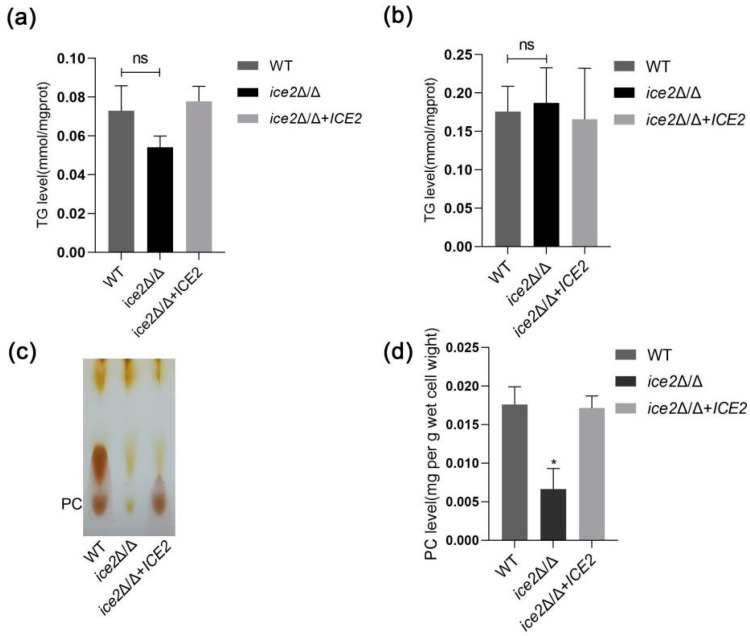
The phosphatidylcholine level decreased in *ice2*Δ/Δ. (**a**,**b**). There is no increase in TG level in *ice2*Δ/Δ. Cells were cultured in SC medium for 6 h in (**a**) and 12 h in (**b**). ns, no significance, *p* < 0.05. (**c**) Phospholipids were separated on silica gel 60 plates. Iodine vapor was used to stain the bands, and the PC band was identified by analyzing the position of PC standards band. (**d**) The intensity of PC band was analyzed using Image J 1.53t, and standard curve was drawn according to the PC band intensity at different standard concentrations of PC after chromatography. PC content was calculated with the standard curve and normalized by cell wet weight. *, *p* < 0.05.

To explain the formation of large LDs, two main hypotheses were postulated: LDs expand via increased neutral lipids or increased LD fusion. For the exploration of the former possibility, we first detected the TG contents in different phases, with the consideration that TGs are the main component of LD-neutral lipids and have been reported to drive LD expansion under extreme conditions [9,36]. The results showed that the TG level did not change in *ice2*Δ/Δ after the cells were cultured for 6 h and 12 h (the time we observed large LDs in *ice2*Δ/Δ) (Figure 3a,b). Therefore, the formation of large LDs is not due to the increase in TG levels.

Next, the other possibility of LD fusion was considered. Phospholipids have been reported to be important regulators of LD fusion. In most mutants with supersized LDs in *Saccharomyces cerevisiae*, especially the mutant of the PC synthesis pathway, PC is believed to suppress LD fusion by reducing the surface tension of LDs [9,37]. Hence, the level of PC was detected with thin-layer chromatography (TLC). The results showed that the PC level in *ice2*Δ/Δ was significantly reduced, indicating that the synthesis of PC is impaired in *ice2*Δ/Δ. This result supports the idea that the emergence of large LDs in *ice2*Δ/Δ is connected to the change in PC level (Figure 3c,d).

Autophagy is also an important mechanism regulating LD growth [11], so we also explored the effect of the *ICE2* deletion on LD autophagy by detecting the degeneration of the LD marker Erg6-GFP [20,30]. The results in Figure S6 suggest that the *ICE2* deficiency inhibits LD autophagy. However, LD autophagy generally occurs after culturing for 3 d in SC medium [30], and we did not detect LD autophagy until the cells were cultured for 5 d. The activation of autophagy only after long-term culturing (5 days) indicated that autophagy is also not involved in the accumulation of large LDs in the mutant at the early period.

### 3.4. Deletion of ICE2 Attenuates the Plasma Membrane Localization of Cho2

**Figure 4:**
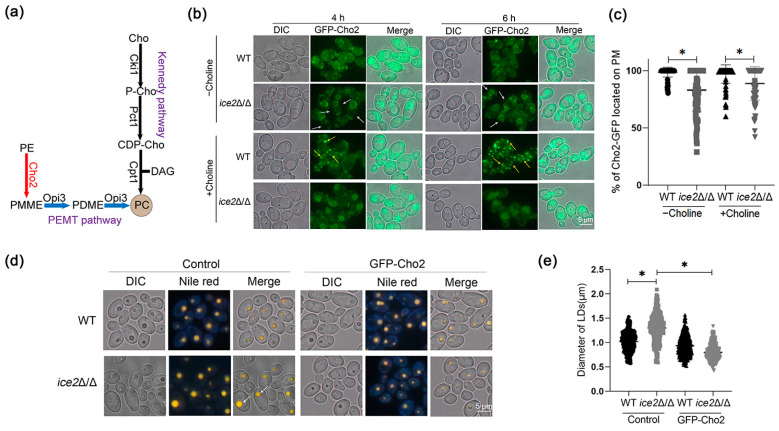
Cho2 has a reduced localization on the PM in *ice2*Δ/Δ. (**a**) Sketch of the two main pathways of PC synthesis in yeast. Cho, choline; P-Cho, phosphocholine; CDP-Cho, cytidine diphosphatecholine; DAG, diacylglycerol; PC, phosphatidylcholine; PDME, phosphatidyldimethylethanolamine; PMME phosphatidylmonomethylethanolamine; PE, phosphatidylethanolamine. (**b**) GFP-Cho2 localization in the WT and *ice2*Δ/Δ strains. The WT and *ice2*Δ/Δ cells were grown in SC medium with 1 mM choline or without choline for fluorescence microscopy. The white arrows denote the PM-localizing defects of GFP-Cho2, and the yellow arrows denote GFP-Cho2 aggregates. (**c**) The percent of Cho2-GFP located on the PM in the individual WT and *ice2*Δ/Δ cells cultured in SC medium for 4 h. The percentage was obtained from the length of the fluorescence signal of cho2-GFP on the PM over the total length of the PM; the length of cho2-GFP fluorescence signal and PM were measured by drawing a curve around the PM and then calculating the length using Image J 1.53t. For each sample, more than 100 cells were assessed. *, *p* < 0.05. (**d**) Overexpression of Cho2 leads to the disappearance of large LDs in *ice2*Δ/Δ. The WT and *ice2*Δ/Δ cells were cultured in SC medium for 12 h. The white arrows denote large LDs. (**e**) LD diameters in the cells grown in SC medium for 12 h. The LD diameters were measured using Image J 1.53t software, and for each strain, at least 300 LDs were measured. *, *p* < 0.05.

As the PC level was largely reduced in *ice2*Δ/Δ, it was important to figure out how they are regulated. In yeast, there are two ways to synthesize PC: the Kennedy pathway (also known as the CDP-choline pathway) and the phosphatidylethanolamine N-methyltransferase (PEMT) pathway (Figure 4a). In the Kennedy pathway, choline is required for initiation, and then it is converted to phosphocholine by choline kinase. Phosphocholine can further be metabolized to CDP-choline by the rate-determining enzyme CTP:phosphocholine cytidylyltransferase, and finally, CDP-choline is transferred to PC by cholinephosphotransferase [38,39,40,41]. This pathway is conserved in different organisms and is considered the main pathway to synthesize PC [42]. In the PEMT pathway, PC is derived from PE via a three-step reaction. The first step is catalyzed by the phosphatidylethanolamine methyltransferase (PEMT) Cho2, and the last two steps are catalyzed by the methylene-fatty-acyl-phospholipid synthase Opi3 [38]. Both Cho2 and Opi3 are important for a normal LD morphology; the absence of either of them can cause supersized LDs [9]. After the observation that the addition of choline eliminated the large LD phenotype in *ice2*Δ/Δ, suggesting that the Kennedy pathway may not be affected, we only focused on the PEMT pathway. Firstly, we labeled Cho2 with GFP to observe its localization. In our results, Cho2 has localization on the PM in both WT and *ice2*Δ/Δ, but PM localization is less pronounced in *ice2*Δ/Δ (Figure 4b,c and Figure S7). This result indicates that the decrease in PC level in *ice2*Δ/Δ may be connected to the changes in Cho2 localization and the decrease in PM localization may be the result of ER–PM contact site reductions. We then observed the LD morphology after Cho2 overexpression in WT and *ice2*Δ/Δ and found that the overexpression of Cho2 resulted in the disappearance of large LDs in *ice2*Δ/Δ (Figure 4d,e), indicating that the accumulation of large LDs in *ice2*Δ/Δ was also connected to the change in Cho2, which further supports our above hypothesis.

In addition, with the supply of choline, Cho2 aggregated abnormally in the logarithmic phase in WT cells, which was not observed in *ice2*Δ/Δ (Figure 4b), suggesting that there may be some regulation of Cho2 with choline supplementation, but *ICE2* deficiency blocked this process. Then, we added PC to the medium [43] and observed the location of GFP-Cho2. The results showed that PC addition can also cause Cho2 aggregation (Figure S8), which proved that Cho2 aggregation is regulated by the PC level.

### 3.5. The Addition of Choline Reduces the Accumulation of Large Lipid Droplets in the ice2Δ/Δ Mutant

**Figure 5:**
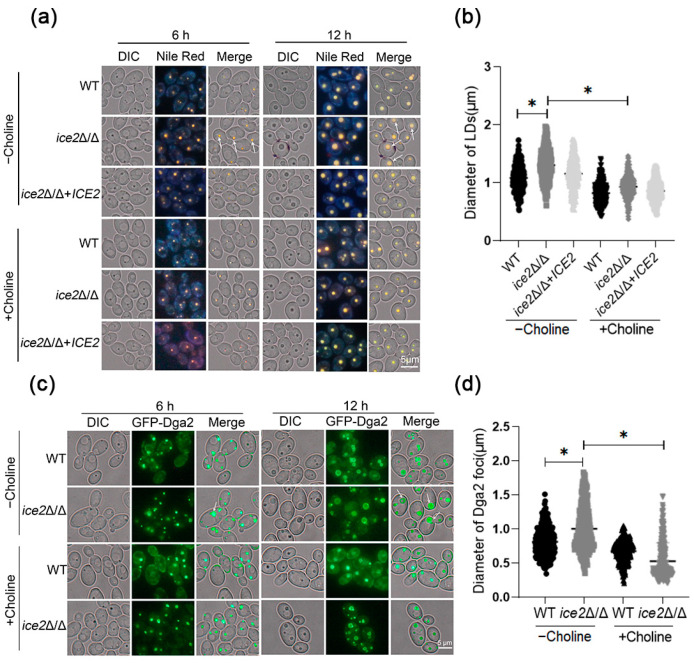
Choline addition leads to the disappearance of large LDs in *ice2*Δ/Δ. (**a**) LDs in WT, *ice2*Δ/Δ, and *ice2*Δ/Δ + *ICE2* cells grown in SC medium with 1 mM choline or without choline were stained with Nile red for fluorescence microscopy. The white arrowheads denote large LDs. (**b**) The diameter of LDs from WT, *ice2*Δ/Δ and *ice2*Δ/Δ + *ICE2* cells grown in SC medium with 1 mM choline or without choline for 12 h. The LD diameter was measured using Image J 1.53t, and for each strain, at least 300 LDs were measured. *, *p* < 0.05. (**c**) The localization of the LD marker GFP-Dga2. WT and *ice2*Δ/Δ cells were grown in SC medium with 1 mM choline or without choline for microscope fluorescence microscopy. The white arrowheads denote large Dga2 foci. (**d**) The diameter of GFP-Dga2 foci from WT, *ice2*Δ/Δ, and *ice2*Δ/Δ + *ICE2* cells grown in SC medium with 1 mM choline or without choline for 12 h. The diameter of GFP-Dga2 foci was measured using Image J 1.53t, and for each strain, at least 300 foci were measured. *, *p* < 0.05.

To confirm the connection between PC level decreases and LD size enlargement, we added choline to the culture and observed the LDs with Nile red staining or Dga2 labeling. The results showed that the accumulation of large LDs was remarkably reduced with the addition of choline, similar to the Dga2 foci (Figure 5), which demonstrated that the reduction in PC level is an important factor in the formation of large LDs. Considering that PC reduces membrane tension, it was supposed that the change in PC level exacerbated LD fusion. In this case, there should be more LDs in WT cells. Therefore, we calculated the number of LDs and observed the LDs after the cells were cultured for 24 h. The results showed that after 12 h and 24 h, there were more LDs in WT compared to *ice2*Δ/Δ (Figure S9), supporting our hypothesis of LD fusion in *ice2*Δ/Δ. Then, the discovery of LD fusion in *ice2*Δ/Δ cells when they were transferred from a choline-rich medium to a choline-free medium provided more evidence (Figure S10). To further analyze the role of choline, we observed the effect of choline addition on ER morphology (Figure S2). It was found that choline addition did not change the ER morphology of WT and *ice2*Δ/Δ, indicating that choline affected LDs independent of the ER. In addition, the growth curve shows that choline addition promoted the growth rate of *ice2*Δ/Δ while restoring the LD phenotype.

## 4. Discussion

ER–PM tethering proteins work as the connecting intermediate between the ER and the PM and are supposed to be involved in substance exchange between the ER and the PM. It has been reported that they are also involved in lipid mobilization, suggesting that they are connected to lipid metabolism [31]. However, there are only a few studies on the regulation of ER–PM tethering proteins on lipid metabolism, especially on the regulation of LDs, the key organelles of lipid metabolism, and the regulation mechanism is not clear. In yeast, there are seven ER–PM tethering proteins: Tcb1-3, the lipid-binding ER tricalbins, which are involved in phosphatidylinositol dephosphorylation regulation, ER-to-Golgi ceramide transport, and ER membrane organization; Ist2, a member of the TMEM16-anoctamin family, which mediates phosphatidylserine transport through coordination with Osh6 and Osh7; Scs2 and Scs22, homologs of the vesicle-associated membrane protein (VAMP)-associated protein (VAP), which interact with FFAT motifs in Opi1p, Swh1p, Osh2p, and Osh3p; and Ice2, a member of the SERINC superfamily, which affects the inheritance of the cortical ER by affecting phospholipid synthesis [32,44,45,46,47,48,49,50,51]. In this study, it was proven that the ER–PM tethering protein Ice2 plays an important role in LD size regulation, as the *ICE2* deficiency led to the accumulation of large LDs in *C. albicans*. This provides evidence that ER–PM tethering proteins regulate the LD phenotype, which enriches the regulatory mechanism of lipid metabolism in fungi. It is regrettable that it is not clear how Ice2 regulates LD size. Moreover, the function of other ER–PM tethering proteins in the regulation of LD metabolism remains to be investigated.

Phospholipids are the main components of biological membranes and are crucial for the maintenance of membrane integrity. These components play important roles in signal transduction, protein structure/function regulation, and specific protein receptor recruitment, which further regulates the morphology and function of different organelles [52,53,54,55,56,57]. For LDs, being special storage organelles, phospholipids form a monolayer on the surface, regulating the stability of LDs by affecting the surface tension and membrane elasticity [58,59,60]. A change in phospholipid content and proportion will have a significant effect on LD morphology. For example, with an increase in the proportion of phosphatidylethanolamine (PE), cholesterol, diacylglycerol (DAG), or fatty acids (FAs), LDs are more likely to fuse into a larger one, while PC has the opposite effect [59,60,61]. In our study, we provided additional evidence that LD morphology is regulated by phospholipids in *C. albicans*. As in the *ice2*Δ/Δ mutant with large LDs, the PC level was greatly reduced compared to the wild type, and the large LDs disappeared with the addition of the PC precursor choline, which increased the PC level through the Kennedy pathway. However, it is not clear if there are also other phospholipids involved in the formation of large LDs in the *ice2*Δ/Δ mutant. In most *S*. *cerevisiae* mutants with large LDs, more than one type of phospholipid changes. For example, in mutants with PC synthesis deficiencies, including *opi3*Δ/Δ, *cho2*Δ/Δ, and *ino4*Δ/Δ, the presence of large LDs is accompanied by decreased PC level and increased PE and PA levels, and adding inositol to improve the overall level of phospholipids could reduce the accumulation of large LDs [9]. These results suggest that there may be a variety of phospholipids involved in LD size regulation. Therefore, analyzing the changes in other phospholipids and exploring their regulation of LD size in *ice2*Δ/Δ are important to elucidate the effect of phospholipids on LDs.

ER–PM tethering proteins have been reported to affect PC level in yeast [31], but the mechanism is not clear. This study showed the phosphatidylethanolamine methyltransferase Cho2 in the PEMT pathway was involved in this regulatory process. In the *ice2*Δ/Δ mutant with decreased PC level, Cho2 reduced the localization in the PM, which led to the hypothesis that a change in the Cho2 distribution may affect its function and thus lead to a PC synthesis deficiency. However, how this change in distribution affects the function of Cho2 is not clear. It has been reported that Cho2 can be phosphorylated by the DNA damage response (DDR) pathway kinase Rad53 [62]. This leads to speculation that changes in Cho2 localization might affect its phosphorylation status and therefore impact its function, as phosphorylation is an important factor in the activity of many enzymes. As for the mechanism underlying the effect of Ice2 on Cho2 localization, it can be speculated that it may be related to the function of Ice2 as an ER–PM tethering protein; the ER–PM contact sites formed with the help of Ice2 may be important for the migration of Cho2 from the ER to the PM. In addition to localization changes, it was found that Cho2 never aggregates in *ice2*Δ/Δ, which was observed in WT cells when choline was added (Figure 4b). The biological meaning of Cho2 aggregation and the mechanisms of Ice2 and PC in the regulation of aggregation are not clear. Further investigation is needed to solve these problems. To summarize, this study can provide some evidences for the mechanism of PC-level regulation via ER–PM tethering proteins, but more studies are needed to verify some of the hypotheses mentioned above.

*C. albicans* is an important opportunistic pathogen. It is of great significance to explore the mechanism underlying its pathogenesis and drug resistance for the prevention and treatment of candidiasis. Therefore, we also investigated the influence of *ICE2* deletion on the pathogenesis and sensitivity to anti-fungal drugs against *C*. *albicans*. The results suggested that *ICE2* deletion did not affect morphogenesis, but did affect the sensitivity to azole and seemed to be related to the change in sterol distribution [63] (Figures S11 and S12). However, the related mechanisms need to be further explored, and an analysis of these mechanisms can provide new evidence for the role of lipid metabolism regulation in fungal drug sensitivity.

In summary, our study uncovered a new function of the ER–PM tethering proteins in LD size regulation. The following mechanism was revealed: with a change in the distribution of the phosphatidylethanolamine methyltransferase Cho2, the synthesis process of PC synthesis is affected, further leading to the alterations in LD size and providing more evidence that ER–PM tethering proteins regulate lipid metabolism.

## Data Availability

Data are contained within the article and supplementary materials.

## Supplementary Materials

The following supporting information can be downloaded at: https://www.mdpi.com/article/10.3390/jof10010087/s1. Table S1: Strains used in this study; Table S2: Plasmids used in this study; Table S3: Primers used in this study; Figure S1: Conserve domain and sequence of *Ca*Ice2. (a) *Ca*Ice2 belongs to the *ICE2* superfamily and contains conserved Ice2 domain, (b) Protein sequence alignment result of *Ca*Ice2 with homologous proteins of Ice2 superfamily; Figure S2: Addition of choline had no impact on ER morphology; Figure S3: Long axis length of cells of WT and *ice2*Δ/Δ cultured in SC medium for 6 h and 12 h; Figure S4: Growth curve of WT, *ice2*Δ/Δ and *ice2*Δ/Δ+*ICE2* with and without choline addition; Figure S5: LDs in hyphae of WT, *ice2*Δ/Δ and *ice2*Δ/Δ+*ICE2;* Figure S6: Deficiency of ICE2 inhibits LD autophagy. (a) Western blots indicating the degradation of Erg6-GFP, (b) The ratio of free GFP to Erg6-GFP. *, *p* < 0.05; Figure S7: Co-localization of GFP-cho2 and PH3-RFP; Figure S8: Addition of choline had no impact on ER morphology; Figure S9: LD morphology at the later stage and statistical analysis of LD numbers in the tested strains. (a) LD morphology of the WT, *ice2*Δ/Δ and *ice2*Δ/Δ+*ICE2* cells cultured in SC medium for 24 h, (b) Number of LDs in more than 300 individual cells of WT, *ice2*Δ/Δ and *ice2*Δ/Δ+*ICE2* cultured in SC medium for 6 h, 12 h and 24 h; Figure S10: Dynamic LD fusion in the *ice2*Δ/Δ cells; Figure S11: Deletion of *ICE2* has no impact on morphogenesis of *C. albicans*. (a) and (b) Deletion of *ICE2* did not affect mycelial development, (c) *ICE2* deficiency did not affect the adhesion of *C. albicans* in vitro, (d) Biomass determination of adhesion cells of C. albicans; Figure S12: Effect of *ICE2* deletion on sterol distribution and sensitivity to azole. (a) Loss of *ICE2* leads to intracellular accumulation of sterols, (b) Loss of *ICE2* leads to the increased sensitivity to fluconazole and itraconazole.
