# Supplementary material for: The Endoplasmic Reticulum–Plasma Membrane Tethering Protein Ice2 Controls Lipid Droplet Size via the Regulation of Phosphatidylcholine in Candida albicans
Source: J Fungi (Basel). 2024 Jan 22;10(1):87. doi: 10.3390/jof10010087 (PMC10817647; doi:10.3390/jof10010087)
Supplement: Supplementary file 1 [file jof-10-00087-s001.zip › jof-2762985-supplementary.pdf]

Table S1. Strains used in this study

Strains	Genotype
WT (BWP17)	ura3Δ::λimm434/ura3Δ::λimm434 his1::hisG/his1::hisG arg4::hisG/arg4::hisG
ice2Δ/Δ	ura3Δ::λimm434/ura3Δ::λimm434 his1::hisG/his1::hisG arg4::hisG/arg4::hisG ice2::ARG4/ ice2::dpl200
ice2Δ/Δ+*ICE2*	ura3Δ::λimm434/ura3Δ::λimm434 his1::hisG/his1::hisG arg4::hisG/arg4::hisG ice2::ARG4/ ice2::dpl200, pDDB78-ICE2
WT+Ice2-GFP Erg6-RFP	ura3Δ::λimm434/ura3Δ::λimm434 his1::hisG/his1::hisG arg4::hisG/arg4::hisG act1:: PACT1-ICE2-GFP-URA3 his:: pDDB78-ERG6-RFP
WT+Ice2-GFP PH3-RFP	ura3Δ::λimm434/ura3Δ::λimm434 his1::hisG/his1::hisG arg4::hisG/arg4::hisG act1:: PACT1-ICE2-GFP-URA3 his:: pDDB78-PH3-RFP
WT+GFP-Dga2	ura3Δ::λimm434/ura3Δ::λimm434 his1::hisG/his1::hisG arg4::hisG/arg4::hisG act1:: PACT1-GFP-DGA2-URA3
*ice2*Δ/Δ+GFP-Dga2	ura3Δ::λimm434/ura3Δ::λimm434 his1::hisG/his1::hisG arg4::hisG/arg4::hisG ice2::ARG4/ ice2::dpl200 act1:: PACT1-DGA2-GFP-URA3
WT+GFP-Cho2	ura3Δ::λimm434/ura3Δ::λimm434 his1::hisG/his1::hisG arg4::hisG/arg4::hisG act1:: PACT1-GFP-CHO2-URA3
*ice2*Δ/Δ+GFP-Cho2	ura3Δ::λimm434/ura3Δ::λimm434 his1::hisG/his1::hisG arg4::hisG/arg4::hisG ice2::ARG4/ ice2::dpl200 act1:: PACT1-CHO2-GFP-URA3
WT+Erg6-GFP	ura3Δ::λimm434/ura3Δ::λimm434 his1::hisG/his1::hisG arg4::hisG/arg4::hisG act1:: PACT1-ERG6-GFP-URA3
*ice2*Δ/Δ+Erg6-GFP	ura3Δ::λimm434/ura3Δ::λimm434 his1::hisG/his1::hisG arg4::hisG/arg4::hisG ice2::ARG4/ ice2::dpl200 act1:: PACT1-ERG6-GFP-URA3
*ice2*Δ/Δ+*ICE2*+Erg6-GFP	ura3Δ::λimm434/ura3Δ::λimm434 his1::hisG/his1::hisG arg4::hisG/arg4::hisG ice2::ARG4/ ice2::dpl200, pDDB78-ICE2 act1:: PACT1-ERG6-GFP-URA3
WT[a]	URA3/ura3Δ::limm434 his1::hisG/his1::hisG arg4::hisG/arg4::hisG
*ice2*Δ/Δ[a]	URA3/ura3Δ::limm434 his1::hisG/his1::hisG arg4::hisG/arg4::hisG ice2::ARG4/ice2::dpl200

Table S2. Plasmids used in this study

Plasmids	Genotype
pRS-ArgΔ*Spe* I	Amp^R ARG4
pDDB57	Amp^R URA3
pGFP-URA3	Amp^R GFP-*URA3*
pDDB78-Erg6-RFP	Amp^R Erg6-RFP *HIS1*
pDDB78-PH3-RFP	Amp^R PH3-RFP *HIS1*
pAU34M-GFP-Dga2	Amp^R GFP-Dga2 *URA3*
pAU34M-GFP-Cho2	Amp^R GFP-Cho2 *URA3*
pAU34M-Ice2-GFP	Amp^R Ice2-GFP *URA3*
pAU34M-Erg6-GFP	Amp^R Erg6-GFP *URA3*

Table S3. Primers used in this study

Name	Sequence (5’-3’)
ICE2-5DR	TCTTTTTGATTCTTTCAGTTCCATCACTCACATGTTTATATATTATTTCCATTTATCA TCTTTCCCAGTCACGACGTT
ICE2-3DR	ATCATTACTCAGTAATTCAGCAGCATAAAGTATTAATGTTGTTCCCATATTAATCCA ATTTGGAATTGTGAGCGGATA
ICE2-5DET	CACAACAATATTTTAGTATT
ICE2-3DET	CTACATTATCCTTTAGTAAT
ICE2-5com	TCCCCCGGGCAATAATGAAGATATTTACA
ICE2-3com	ATTTGCGGCCGCAGCATTAAATTAATAACTAA
ICE2-5inner-1,2	TGTTTAGTGCTTCGTTGTTGGGA
ICE2-3inner-1	AACTTGACGTGAACCTGGTTGAG
ICE2-3inner-2	GGTTGAGGAGTTTTTGGTGGTGT
URA3-5inner	CGCGGGATTTGGATGGTAT
URA3-3inner	TCTTGGCTCTTGGTTGGTG
URA3-5DET	GCCAGTGAATTGTAATACGAC
URA3-3DET	TCACACAGGAAACAGCTATGA
ICE2-5GFP	CCGCTCGAGATGCCCATAATTAAAACTAT
ICE2-3GFP	TCCCCCGGGTTCTACTTTCCAATGACTAG
DGA2-5GFP	TCCCCCGGGATGACAGACACATCTGACCT
DGA2-3GFP	CGCGGATCCTTATTCGACGATACTGAATT
Erg6-5RFP	CTAGCTAGCTAGATGTCTCCAGTTCAATTAGC
Erg6-3RFP	TCCCCCGGGGGAATCTTTCTTTTCTAATGGTT
CHO2-5GFP	TCCCCCGGGATGACTTTCACAATCAATAA
CHO2-3GFP	CGCGGATCCTTATACATCTTTTTTATCAG

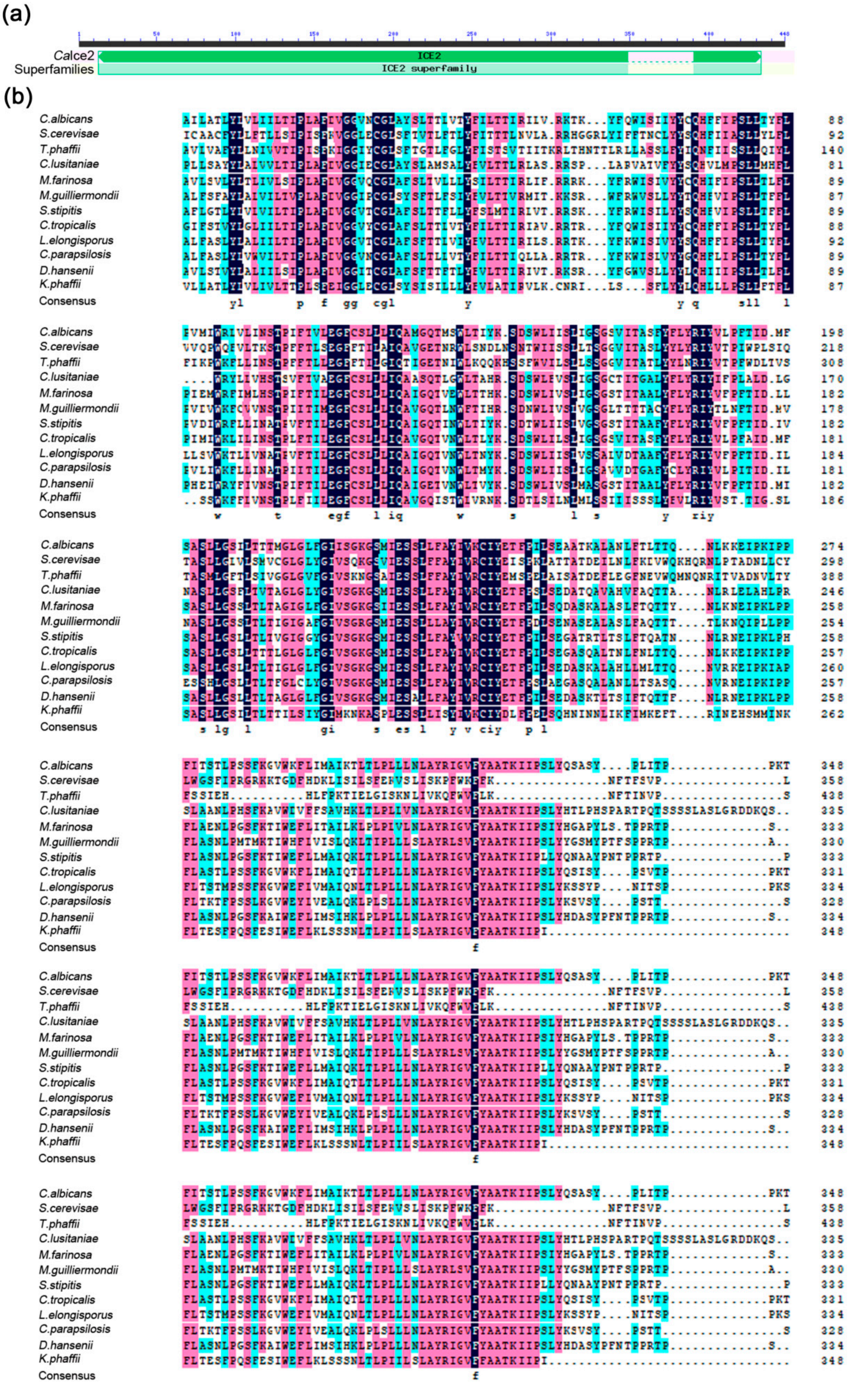

Figure S1. Conserve domain and sequence of *Ca*Ice2. (a) *Ca*Ice2 belongs to the *ICE2* superfamily

and contains conserved Ice2 domain. Domain architecture ID: 10552640. (b) Protein sequence alignment result of *Ca*Ice2 with homologous proteins of Ice2 superfamily. The species to which the sequence belongs are shown on the left.

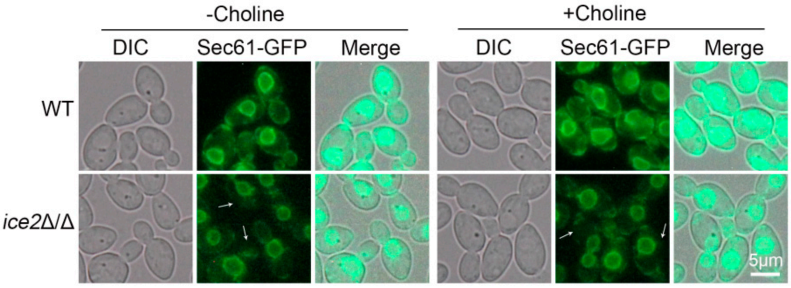

Figure S2. Addition of choline had no impact on ER morphology. Sec61-GFP: marker of ER. Cells were cultured in SC medium with 1 mM choline or without choline for 5 h. White arrow denote cortical ER deficiency.

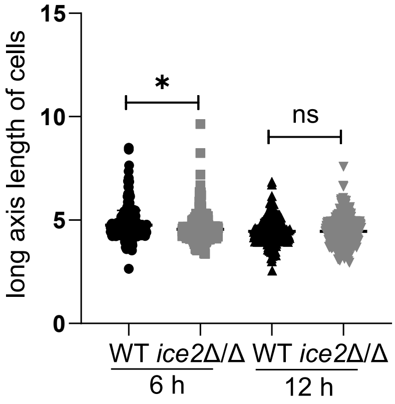

Figure S3. Long axis length of cells of WT and *ice2*Δ/Δ cultured in SC medium for 6 h and 12 h. One-way ANOVA method was used for statistical analysis, ns, no significance,*, $p < 0.05$.

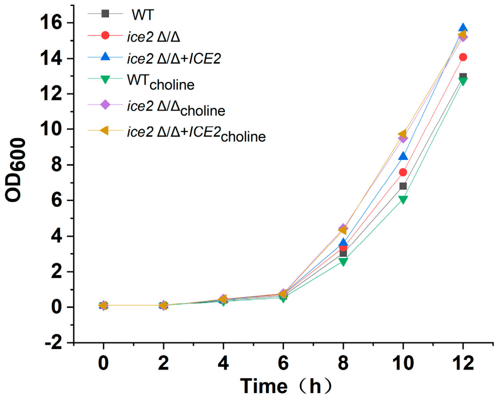

Figure S4. Growth curve of WT, *ice2*Δ/Δ and *ice2*Δ/Δ+*ICE2* with and without choline addition. All strains were cultured in SC medium at a constant temperature of 30°C and 160 rpm.

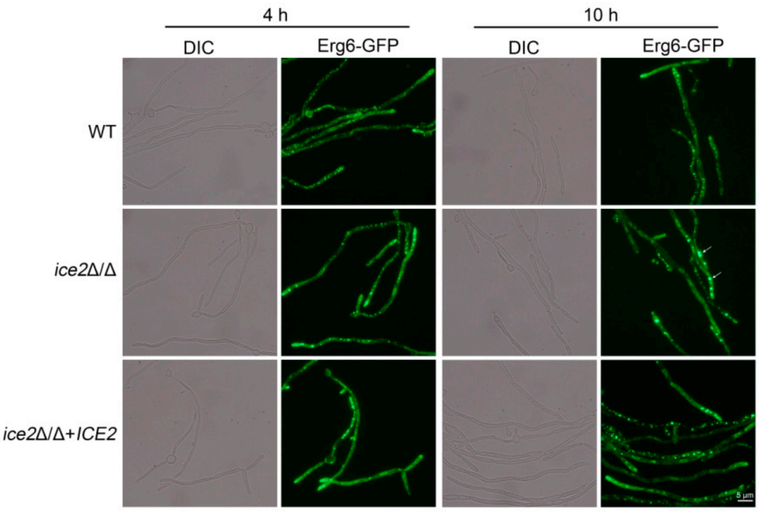

Figure S5. LDs in hyphae of WT, *ice2*Δ/Δ and *ice2*Δ/Δ+*ICE2*. The cells were cultured in the RPMI 1640 medium at 37 ℃ for 4 h and 10 h, respectively, followed by fluorescence microscopy. Erg6-GFP, the marker of LD. The white arrows indicate the LD aggregates.

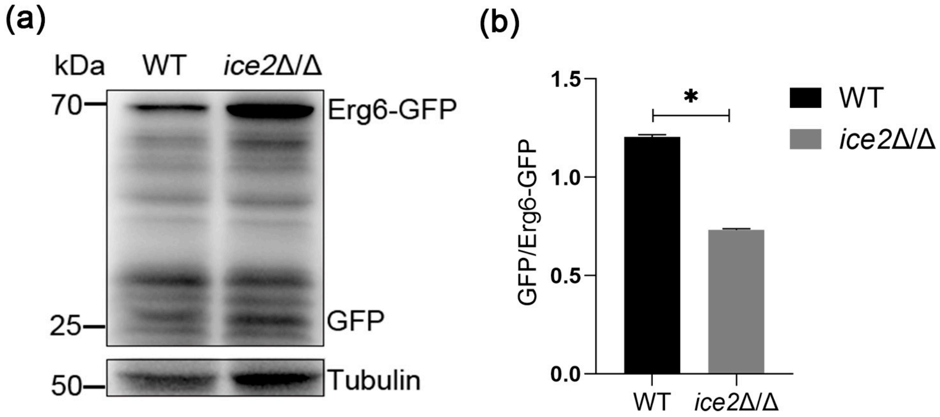

Figure S6. Deficiency of *ICE2* inhibits LD autophagy. (a) Western blots indicating the degradation of Erg6-GFP. Erg6-GFP, the marker of LD. The cells were cultured in SC medium for 5 d, followed by Western blotting. (b) The ratio of free GFP to Erg6-GFP. *, $p < 0.05$.

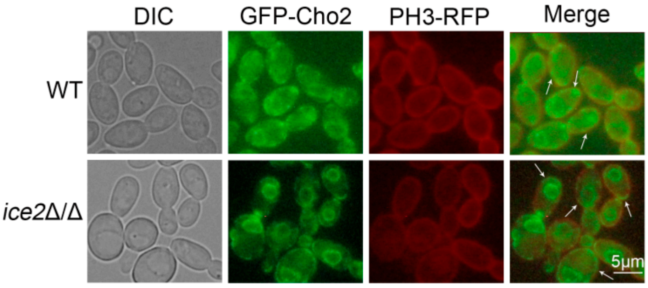

Figure S7. Co-localization of GFP-cho2 and PH3-RFP. PH3-RFP: marker of the PM. The white arrows denote localization of GFP-Cho2 on the PM.

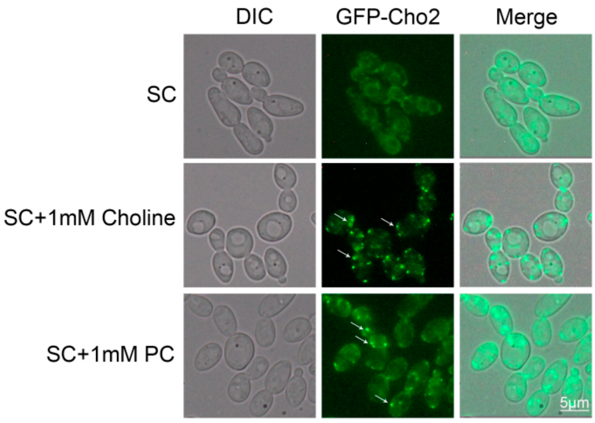

Figure S8. Addition of choline had no impact on ER morphology. Sec61-GFP: marker of ER. Cells were cultured in SC medium with 1 mM choline and without choline for 5 h. White arrow denote cortical ER deficiency.

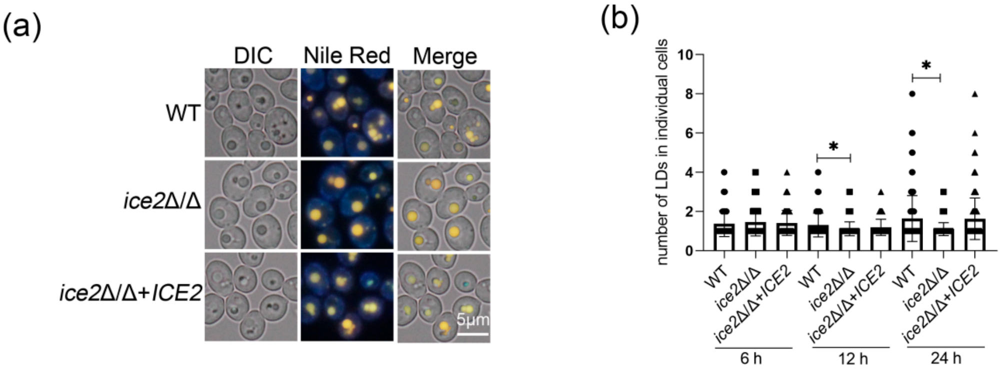

Figure S9. LD morphology at the later stage and statistical analysis of LD numbers in the tested strains. (a) LD morphology of the WT, *ice2*Δ/Δ and *ice2*Δ/Δ+*ICE2* cells cultured in SC medium for 24 h. (b) Number of LDs in more than 300 individual cells of WT, *ice2*Δ/Δ and *ice2*Δ/Δ+*ICE2* cultured in SC medium for 6 h, 12 h and 24 h. One-way ANOVA was used for statistical analysis, *, $p < 0.05$.

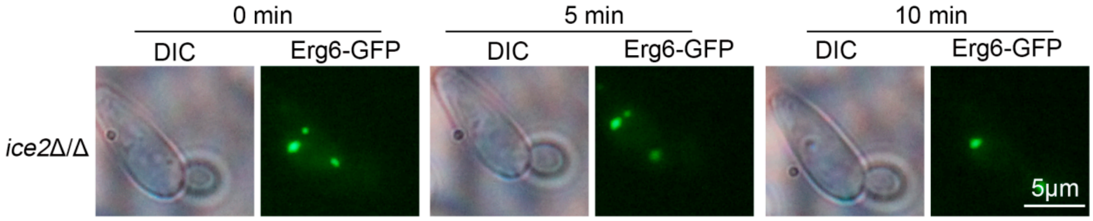

Figure S10. Dynamic LD fusion in the *ice2*Δ/Δ cells. The cells were cultured in the choline-rich SC medium for 5 h and then transferred to the choline-free SC medium for further observation. Erg6-GFP: the marker of LD.

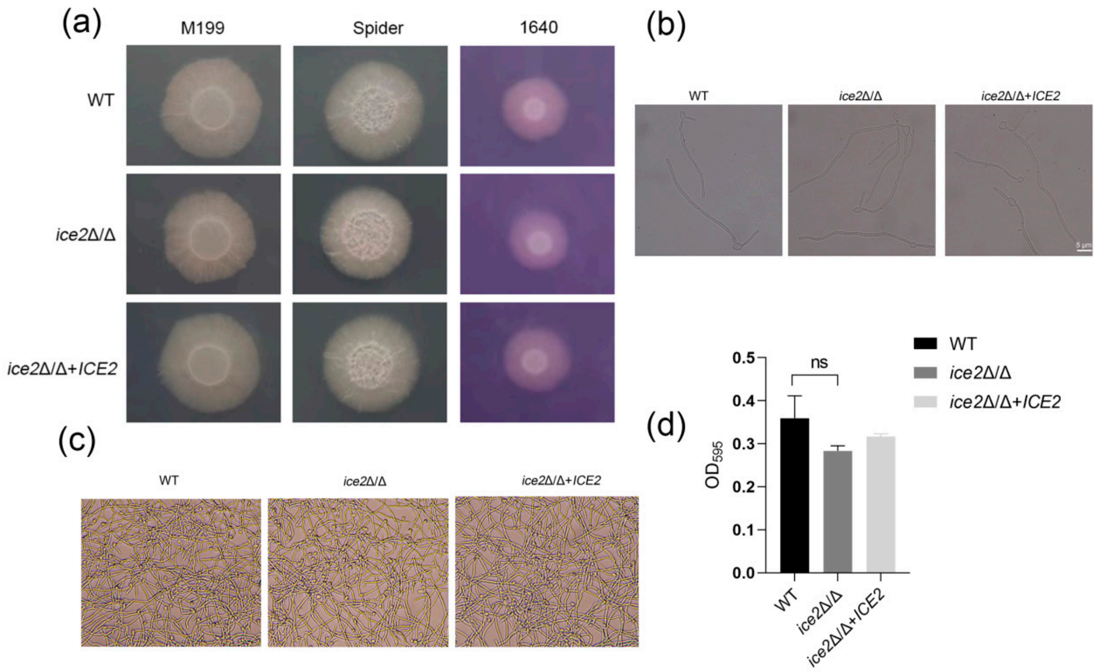

Figure S11. Deletion of *ICE2* has no impact on morphogenesis of *C. albicans*. (a) and (b) Deletion of *ICE2* did not affect mycelial development. For solid hyphal induction, 3 μL cell solution of WT, *ice2*Δ/Δ and *ice2*Δ/Δ+*ICE2* with OD_{600} of 0.3 were added to M199, Spider and 1640 solid medium and cultured at 37 ℃ for 5 d. For liquid hyphal induction, cells were culture in 1640 medium at 37 ℃ for 4 h. (c) *ICE2* deficiency did not affect the adhesion of *C. albicans* in vitro. The polyethylene porous plate was used for adhesion in vitro. (d) Biomass determination of adhesion cells of *C. albicans*. The adherent cells stained with crystal violet were washed from the polyethylene pore plate with glacial acetic acid, and the supernate was taken to measure OD_{595}, ns, no significance.

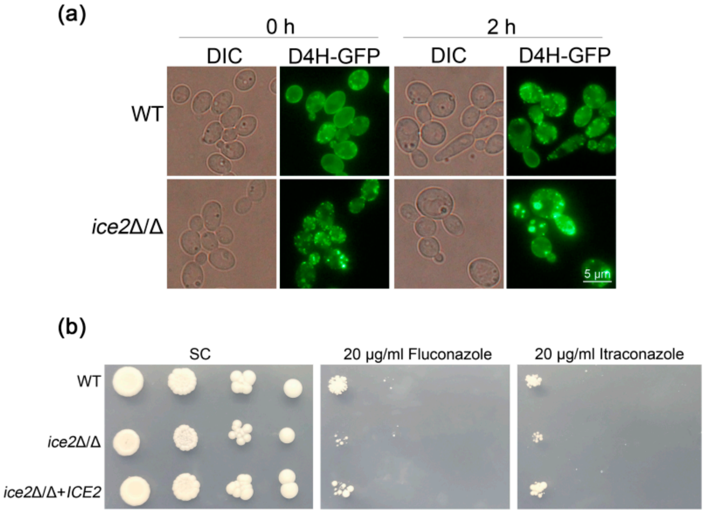

Figure S12. Effect of *ICE2* deletion on sterol distribution and sensitivity to azole. (a) Loss of *ICE2* leads to intracellular accumulation of sterols. D4H-GFP[63], the marker of sterols. Cells were cultured in SC medium. (b) Loss of *ICE2* leads to the increased sensitivity to fluconazole and itraconazole.
